# Buffalo Clinical and Pathological Society

**Published:** 1889-09

**Authors:** 


### THE BUFFALO CLINICAL AND PATHOLOGICAL SOCIETY.

Regular meeting, July 19, 1889.

The subject of Cholera Infantum was discussed.

### ETIOLOGY AND MORTALITY OF CHOLERA INFANTUM.

BY IRVING M. SNOW, M.D.

I WAS requested, a few days ago, to write upon the etiology and mortality of acute intestinal diseases of children, and wishing very much to ascertain how important a role cholera infantum played in the vital statistics of Buffalo, I have diligently studied the reports of the local board of health, for the past three years, seeking to discover the influence of our Summer climate upon young children, and also endeavoring to discover the prevalence of the disease in different wards of the city, the influence of nationality, and of wealth and poverty.

Wasserfuhr, an eminent German authority, states that when the proportion of children born alive who die in their first year exceeds nineteen per cent., the mortality is excessive, and should awaken alarm and vigilance in the community. Bearing this assertion in mind, I have turned to the vital statistics of Buffalo, with the following result:

In 1886, the proportion of children dying in their first year was 19.4 per cent.; in 1887, 21.2 per cent., and in 1888, 22.5 per cent.

Therefore, in Buffalo, the infant mortality is excessive, and is evidently steadily increasing.

Remembering the healthful location of our city, which is not densely populated in any quarter, and the comfortable circumstances of nearly all its citizens, I have again analyzed the vital statistics to discover the cause of the great infant mortality.

Only five per cent. of children under one year die of contagious diseases, only ten per cent. of respiratory diseases, and nearly one-third or thirty-one per cent. of acute intestinal diseases. Thus, the cause of the very large death rate among infants is from acute diarrheal diseases. A very striking difference is to be found between the hot and cool seasons of the year. The average mortality for 1886–'87–'88 among children under three years in the twelve hot weeks, was fifty-four per week, while for the remainder of the year, forty weeks, only twenty children died per week. I therefore find that the excessive mortality among young children is caused by acute intestinal diseases, prevailing in the twelve hot weeks of the year. After diligent study of the vital statistics of Buffalo for 1886–'87–'88, I have prepared a number of tables showing the progress of diarrheal disease among young children through the various weeks of the Summer, the average temperature and humidity, and also the distribution of cholera infantum in the various wards. I will only read the results of my investigations and the conclusions which must be drawn from them.

The total number of deaths among children under three years from cholera infantum, diarrhea, and dysentery was: in 1886, 387; 1887, 515; 1888, 567; showing a steady increase in three years. Although the city is steadily growing in population, I do not believe that the increased death-rate among children is sufficiently accounted for by the greater number of inhabitants. May not this difference be due to a variation in our Summer climate, as it is a well-known fact that one Summer is often warmer than the next. Excessive heat, or a high average temperature during the hot season, does not necessarily cause a large infant mortality from intestinal troubles. Thus, in July, 1886, the mortality was 158, the average temperature being 69.4° F., but in July, 1887, the mortality was 265, 117 deaths more, while the average temperature was 68.4°, one degree cooler. In July, 1887, the number of deaths returned from acute intestinal troubles was 265; in August, the same year, only 168 deaths were returned, ninety-seven less, yet August, the month of the lower mortality, the average tem perature was 6.2° greater.

The mortality in Buffalo follows the line of minimum temperature with great regularity. Thus, when the minimum temperature is high (hot nights), the death-rate is high; when the minimum temperature is low (cool nights), the mortality is least. The death-rate from cholera infantum in Buffalo is usually greater in July than in August, but only when steady heat prevails, the variation in temperature between night and day being usually greater in August than in July. This is easily proven by comparing the death-rate and minimum temperature for the various months. For July, 1886, the minimum temperature was higher (59.1°), and the mortality higher (148) than in August, 143, 53.1°. Also in 1887, in July, the minimum temperature was 67.5°, number of deaths 265, while in August, when the minimum temperature was low, 64.2°, the death-rate was also low (168.) In 1888, August was more unhealthy than July, for the minimum temperature was greater, 59.6° to 64°, in July, while twenty-three more deaths were returned in August than July.

TABLE OF TEMPERATURES FOR JULY AND AUGUST, 1886, 1887, AND 1888,

Showing that the death-rate in children from acute intestinal diseases does not depend upon the heat of the Summer, but follows the minimum (night) temperature with great regularity :

| | | Deaths. | Average Temperature. | Average Minimum Temperature. |
|---|---|---|---|---|
| 1886 | July | 144 | 69.4° | 60.3° |
| | August | 141 | 67.2° | 60.3° |
| 1887 | July | 265 | 68.4° | 67.5° |
| | August | 168 | 74.6° | 64.2° |
| 1888 | July | 189 | 67.4° | 59.6° |
| | August | 212 | 67.4° | 64.0° |

Seibert, in an article upon cholera infantum read before the New York Academy of Medicine, 1888, noticing that a cool Summer may be more fatal than a hot Summer, reached the conclusion that when the minimum temperature reached 60.1° above, and remained so for a week, cholera infantum becomes epidemic, yet I do not think that this assertion holds true for Buffalo, for, the week ending June 23, 1888, with a minimum temperature of 60°, and an average temperature of 74.3°, returned only twelve deaths, and the next week even less (8), while the week ending July 28th was much cooler, the minimum temperature being 54.5, the average temperature 68.4, yet the mortality was 57°.

As. Dr. Seibert's opinion was reached after a most careful investigation, I believe that a lower minimum or average temperature will produce cholera infantum in Buffalo than in New York or Philadelphia; for, turning to the Philadelphia statistics, the average temperature for seven years for the month of June was about 72°, and the proportion of children dying of acute intestinal diseases, to the total mortality was as 1 : 14½. In Buffalo, for July, the average temperature was only 68.4°, while the number of children dying of acute intestinal diseases to total mortality was as 5 : 14.

My own conclusions agree with those of Turner, of Portsmouth, England, who found that a minimum temperature of 50° F. was sufficient to cause a considerable mortality from cholera infantum.

*Influence of humidity and rain-fall upon prevalence of Cholera Infantum.*—In 1886, a wet season, the average daily humidity was 73.6, and the deaths in twelve weeks were 358. Eighteen hundred and eighty-seven, a dry year, the average daily humidity was 68.5, and the number of deaths was 481. Thus, dry weather seems to favor the prevalence of cholera infantum ; a wet year seems to be a healthier year.

An explanation of this may be that a frequent rain-fall tends to purify the air of bacteria, flushing out sewers and pools of stagnant water, and also by laying dust.

Having dwelt rather hastily upon the effects of temperature and humidity upon young children, I will next consider the influence of density of population. It is a well-known fact that in the same community, and during the same season, the infant mortality shows a great variation in different neighborhoods. Nowhere is this more striking than in Buffalo.

The second ward, with the densest population, sixty-three to the acre, shows the lowest infant mortality. This neighborhood is full of boarding and lodging houses and hotels, and might include fewer children in its population than other wards.

The tenth ward, with a population of thirty-five to the acre, and which contains several large infant asylums, has a much lower death-rate than the fourth ward, with sixty to acre.

The eleventh ward, with thirteen souls to the acre, has a lower infant death-rate than the seventh, with twenty-six to the acre. Thus, the eleventh ward, with about 30,000 people, contributed only 6.3 per cent. of the infant mortality from diarrheal diseases, while the seventh, with 40,000 people, gives sixteen per cent. of the mortality.

Again, the twelfth ward, with less than two people to the acre, and 3.2 per cent. of total population furnishes two per cent. of the deaths from acute intestinal diseases among young children ; while the tenth, with thirty-six people to acre, eighteen times as dense, and 3.6 per cent. of total population, has a lower death-rate, giving only 1.5 per cent. of deaths. Hence, the ravages of cholera infantum in Buffalo are not dependent upon density of population, for the death-rate in a sparely populated district, as the twelfth ward, may be very high, while in a relatively densely populated district as the tenth ward it may be very low. I may even say that the disease may be more fatal in the country than in certain districts of a city, for, in proportion to the population, the death from cholera infantum in the sparsely populated thirteenth ward, is twelve per cent. greater than in the tenth, where the population is thirty-five times denser.

A great difference also exists between the neighborhood inhabited by people in easy circumstances and the districts inhabited by the working classes. Thus the mortality in the ninth and tenth wards, which are inhabited by well-to-do families, is really the lowest in the city. I find that, in proportion to the population, the infantile mortality from cholera morbus, in the fifth ward—inhabited by workingmen of German and Polish extraction—is three and a quarter times greater than the infant mortality from the same disease in the tenth, although the tenth ward has thirty-five people to acre, and the fifth only twenty-one to acre. This surprising difference is greater than the ratio existing in the mortality of the English gentility and the English agricultural laborer. Nevertheless, the proof that a low infant mortality from intestinal diseases may prevail among people of the working class, may be seen by comparing the first with the sixth ward.

The first ward, an Irish district, with 7.5 per cent. of population, and a density of twenty-four to the acre, contributes 5.5 per cent. to the mortality, while the sixth ward, a German district, with 11.3 per cent. of the population, and the same density of population, contributes 15.5 per cent. of the total infant mortality from diarrheal diseases, relatively twice as great. This great contrast in the death-rate among the children of the richer and poorer classes is due to the difference in medical care, in personal hygiene, and in the quality and care of milk.

The fifth ward is populated by German and Polish workingmen, drawn mostly from the villages and rural communities of Germany and Poland, yet the mortality among their children is relatively greater among the children of the well-to-do classes, many of whose parents were born in the city, and are city-bred.

The so-called American wards have the lowest infant mortality from cholera infantum; the Irish wards the next; the German wards are next, while the mortality from cholera infantum in the Polish quarter is extraordinarily great. I, however, do not believe that Polish children are unusually susceptible to diarrheal diseases, or that their vital resistance to disease is low. On the contrary, in my opinion, the great mortality among them is due to their crowded dwellings, and to the unsanitary condition of their surroundings, bad water supply from wells, bad drainage, and to the administration of old and spoiled milk to their children. The so-called Polish quarter is in the fifth ward, which is not densely populated, not more than 2.122 souls to the acre. Yet, with twenty-two per cent. of the population, it contributes one-third (32.5) per cent. of the total deaths from cholera infantum.

In comparing the records of the last three years with Dr. Campbell's table of deaths from diarrheal diseases from 1881 to 1886,[1] I find some striking changes. Keeping always to the mortality of children under three years, of diarrheal diseases, I find that the health of the tenth ward, despite its large infant asylums, has vastly improved. In Dr. Campbell's statistics it ranked sixth in point of health, and returned 5.6 per cent. of the infant mortality from cholera infantum. An average for 1886–'87–'88 shows that now it ranks third in point of health, and contributes only 2.7 per cent., less than half its former proportion, from cholera infantum. A similar improvement exists in the fourth ward district around Main and Genesee—East side. The ward which shows the greatest relative increase is the thirteenth, a ward which I believe has a nearly stationary population. The mortality from cholera infantum has quadrupled in eight years. The ward is sparely populated, the soil is clay, water supply mostly from wells, drainage is bad.

PREVALENCE OF CHOLERA INFANTUM IN DIFFERENT WARDS OF THE CITY, FROM THE VITAL STATISTICS OF 1886, 1887, AND 1888.—WARDS ARRANGED IN ORDER OF DENSITY OF POPULATION.[2]

| Ward. | Percentage of Total Population. | Percentage of Deaths from Cholera Infantum. |
|---|---|---|
| Second | 3 3 | 1.5 |
| Fourth | 4.0 | 2.8 |
| Third | 5 8 | 5.7 |
| Ninth | 3.6 | 1.5 |
| Tenth | 5.8 | 2.7 |
| Eighth | 4.2 | 3.5 |
| Seventh | 15.4 | 16.0 |
| First | 7.5 | 5.4 |
| Sixth | 11.3 | 15.5 |
| Fifth | 20.7 | 32.4 |
| Eleventh | 11.0 | 6.2 |
| Twelfth | 4.2 | 4.8 |
| Thirteenth | 3.2 | 2.0 |

ETIOLOGY.

The conditions which predispose children to cholera infantum are sufficiently well known, and require here no consideration. There is

1. BUFFALO MED. AND SURG. JOURNAL, Vol. xxv., p. 193, *et seq.*

2. It is to be regretted that, in comparing the mortality from cholera infantum in the various wards of the city, the writer could find no statistics giving the relative number of children in each ward. It is, however, certain that there are fewer children in the richer districts (9th and 10th wards), than the wards inhabited by the working class.

much evidence to show that the true cause of acute intestinal diseases of children is bacterial infection, usually of the milk. Children who are suckled seldom fall ill of cholera infantum. Among 1,000 children dying of diarrheal diseases only thirty had been entirely breast-fed. Human milk is absolutely aseptic, and resists bacterial infection for a surprising long period of time. Cow's milk, as sold in cities, always contains bacteria. Booker found twelve varieties in cows' milk. A certain amount of heat is necessary to, and favors bacterial-growth diseases. Thus milk not at once cooled, and kept cold, readily becomes infected and poisonous in Summer.

Children in a New York tenement-house live, virtually, in the same atmosphere Winter and Summer; for in cold weather the windows and doors are kept shut, warmth being procured at the expense of ventilation; yet cholera infantum prevails among them only in Summer. The reason is, that in Winter the milk is kept outside the window, in a cool place, while in Summer it is impossible to keep milk cold.

The greater mortality from diarrheal diseases in Summer is due not to the effect of heat upon the child's body, but upon the child's food. Milk turns sour when exposed to a temperature of 60°. The acidification of milk is due to a microörganism, which is the cause of the lactic acid fermentation, and this is just about the minimum temperature when cholera infantum prevails.

Escherich, of Munich, found that the acid fermentation present in disease of the upper intestinal tract was due to bacteria.

Tompkins, of Leicester, found that the air in quarters of the town infected with cholera infantum loaded with bacteria, while in the quarters of the town where cholera infantum was rare, the atmosphere was comparatively free from microörganisms. He suggests the easy infection of milk in the sickly quarter.

Hayems, an Italian physician, observed that when a child suffering from green diarrhea came into his wards, numbers of previously healthy children were attacked with green diarrhea. Disinfection of the excreta, and isolation, caused the disappearance of the epidemic. His pupil, Lesage, isolated a bacillus which, when injected into the blood, always produced green diarrhea, caused by the multiplication of bacilli. Lesage's experiments have been confirmed by numerous observers. Victor Vaughan has isolated a ptomaine, called tyrotoxicon, from spoiled milk, which he believes is the most frequent cause of cholera infantum. He has found tyrotoxicon in many specimens of milk, which had produced vomiting and purging in adults and children. This ptomaine, if extracted from milk and given to animals, produces the symptoms and pathological changes present in cholera infantum. He found tyrotoxicon in milk taken by a child afterwards attacked by cholera infantum. He believes that the most frequent cause of acute intestinal disease in children is due to changes produced by micrococci in milk exposed to a temperature of over 60° ; that the slight mortality of children suckled at the breast is due to the fact that human milk is free from disease-germs, while cow's milk contains great quantities of bacteria; mucous and catarrhal diarrhea are due to ordinary bacteria, while serous and choleriform diarrheas are due to pathogenic germs which produce a chemical poison ; that this poison is identical with tyrotoxicon, and may be produced by infecting milk with the bacillus of butyric acid fermentation, and keeping it in a tightly-closed vessel from eight to ten days.

He, therefore, in cases of cholera infantum, recommends the immediate withdrawal of milk, which may contain tyrotoxicon, and the substitution of chicken or mutton broth, or albumen water. The existence of the pathogenic effects of tyrotoxicon have been established by many control experiments. Feebly boiled milk contains no tyrotoxicon, the ptomaine being expelled by heat.

May we not, then, regard cholera infantum as a preventable disease, and cannot its ravages be greatly lessened by greater care in the feeding of children ? This is really a very simple matter. It merely involves keeping the milk clean, putting it in a cold place, and sterilizing it by heat before the child takes it. It involves but little time and slight expense, and would, every Summer, save thousands of children from sickness and death.

### SYMPTOMATOLOGY AND PATHOLOGY OF CHOLERA INFANTUM.

BY EUGENE A. SMITH, M. D.

Acute intestinal diseases in children are known under the several names cholera infantum, gastro-enteritis choleriformis, Summer cholera, choleraic diarrhea and Summer complaint. It is defined as an acute gastro-intestinal catarrh in young children, sudden in outset, and of grave prognosis (Conklin). In sickly children, especially those artificially fed, and even among healthy infants, it is not unusual to see in one day the onset of the disease and its progress to a fatal termination.

In diagnosis, it is only to be differentiated from Asiatic cholera and the chronic forms of diarrhea in children known as entero-colitis or Summer diarrhea. The latter trouble differs from cholera infantum in severity, duration and extent of intestinal tract involved; but the line of demarkation is often obscure.

In brief, the symptomatology of cholera infantum includes two distinctive features, namely, large watery stools, and more or less profuse vomiting. Besides these are found high temperature, pain and prostration, and wasting with their attending phenomena. The prodromal stage, when present, is usually short, lasting from a few hours to one or two days. The child may be feverish and restless, refuse to nurse, and suffer from colic. Often, however, diarrhea and vomiting, rapidly becoming grave, announce the developed disease in a seemingly healthy infant. Where errors in feeding induce the sickness, simple vomiting, or diarrheal discharges of undigested food, may precede the more acute symptoms. Cough, with fever, may precede attacks due to exposure and chilling of the surface of the body. Chronic diarrhea, due to entero-colitis, frequently terminates fatally in the development of cholera infantum, and it may also be said that cholera infantum in its progress to recovery often passes into a chronic diarrhea. I have frequently observed that unhealthy movements of the bowels, preceding the disease, are entirely overlooked by mother and nurses. The child may be costive, straining during defecation, or it may have daily three to several bad-smelling, clay-colored passages, containing undigested milk, for some time before more alarming symptoms set in; but, unless particularly questioned in this respect, the mother misleads the physician by stating the child's bowels were regular. Under such conditions, the onset of cholera infantum may be inadequately treated as simple indigestion, or diarrhea from transient irritation.

After the first bad-smelling diarrheal movement of cholera infantum have swept away undigested matters, feces, and intestinal mucus, the following passages are milky and odorless, and later watery, soaking through the clothing and staining the linen a greenish color. The earlier movements are such as occur in the symptomatic diarrhea of indigestion, and are acid in reaction, but the movements, distinctive of the disease, are watery, due to excessive intestinal secretion, the greenish stain being due to small quantities of bile. Pepper states the excessive fluid is not alone secreted from the glands, but exudes as a perspiration or exosmosis from the entire surface of the intestine; and as the intestinal tract in infants, compared to length of body, is longer than in the adult, we can better understand the rapidity and amount of fluid loss. The movements may be frequent, four or five in an hour, or less frequent and more profuse to the extent of justifying the mother, who lately told me her child could swim in its own passages. Peristalsis is least free when cerebral complications induce torpidity by dulling reflex intestinal sensibility. Often in such cases it is hoped that the diarrhea is checked, until a copious exhausting movement reveals the amount of secretion which, like a concealed hemorrhage, insidiously drains the strength of the patient away. Naturally, under these conditions thirst is extreme, and the child will drink or nurse with frantic eagerness, only, however, to reject it again. The urinary secretion is small or suppressed, and it may remain so two or three days, if the profuse diarrhea continues. The skin is dry, because the sweat-glands are inactive from want of fluid. The vomiting is not the "driveling" regurgitation of an overfed infant's stomach, nor the sour, curdled milk of the indigestion and fermentation seen in the early hours. Water, medicine, and food are forcibly ejected, and the acid contents of the stomach are later succeeded by alkaline and bilious intestinal secretions. Vomiting occurs readily in childhood, because the cardiac end of the stomach has less sphincter development, and the stomach does not lie transversely as in the adult, but vertically. Besides this, the pelvis is small and the liver large, making the abdomen prominent, and thus in vomiting a greater expulsive force is applied in emptying the stomach.

The temperature of the surface often seems about normal, but axillary measurement reveals 103–105° F. When it is remembered that children are prone to hyperpyretic temperature upon slight irritation, it is not strange that we find high fever where such extensive intestinal irritation coöperates with absorption of products of fermentation and inflammation. In cases developing a cerebral complication, the extremities may be cold and the surface temperature seem subnormal, but the head is hot, with the veins of the scalp turgid, the fontanels pulsating, and the axillary temperature registering perhaps 105° F.

The pain caused by cholera infantum does not seem commensurate to the gravity of the disease when compared with the agony caused by colic. Infants are prone to colic, owing to the lack of development of the muscular structure of the intestine, thus permitting flatulent distension, which creates muscular spasm. In cholera infantum, however, the pain and tenderness are more due to normal peristalsis in the inflamed intestine than to flatulence and spasm. Many cases, indeed, suffer a minimum of pain, and here *post mortem* examination reveals a minimum of intestinal congestion. When intestinal pain exists, it causes fits of crying, with contortions of the body and flexion of the thighs on the abdomen.

Exhaustion and wasting rapidly develop in cholera infantum. The loss of fluid, the muscular efforts in vomiting, and the failure to nourish, are the potent causes. The tissues lose their tone, the skin and lips become dry, the tongue coated, the fontanels sunken, and the features pinched. Unless suffering from the hyperesthesia of cerebral complications, the child soon sinks into apathy, insensibilities and collapse.

The anatomical changes in cholera infantum are those which develop from intestinal inflammation, and they vary according to the duration of the disease. On July 2, 1889, I made a *post mortem* on a well-nourished, nursing baby, thirteen months old, which had died in a convulsion, following a paroxysm of whooping-cough. The child died at nine P. M., July 1st. At four P. M. I had found it feverish, but bright and strong. The nurse reported a profuse diarrhea, with vomiting, which had lasted eighteen hours, and which she ascribed to "teething." Some time before death the vomiting and diarrhea were stopped by the administration of calomel ½ gr. and bismuth gr. ii., repeated at hourly intervals.

On opening the abdomen I found no more than the usual gaseous distension. The peritoneum seemed normal. The intestines appeared normal, with the exception of a few reddened patches in the ileum and colon. The stomach, examined closely, externally and internally, showed no evidence of morbid change. It contained milk not curdled, diluted by the gastric secretions. The intestines contained a milky, thin secretion, and on the mucous surface over the few reddened spots, a slightly tenacious, watery secretion was more abundant. In several places, aside from the reddened parts, the intestine was injected in small patches, the longest not covering a square inch. The injection was arborescent, the spots being round or elliptical in form, and resembling the enlarged microphotographs of the capillary circulation of follicular glands seen in text-books of physiology. In the reddened patches, the mucous membrane was puffed, softened, and its glands rendered more prominent. In no place was there tissue destruction.

Extremely acute cases of short duration often present still less, or even no macroscopical anatomical changes, but the microscope always shows the beginning of an inflammatory process. The apparently normal condition of the intestinal tract found in these cases has led some writers to remove cholera infantum from the list of intestinal diseases, and place it with sunstroke among the cerebral affections. They ascribe the serous discharges and the vomiting to cerebral changes, and they look upon the more pronounced intestinal changes found in less acute cases as the result rather than the cause of the disease.

In cases which run a longer course, however, as in those which develop in the course of a chronic diarrhea, the macroscopic morbid anatomy is usually more pronounced, approaching that of chronic Summer diarrhea. Rings of congestion about the glandular openings may cause the intestine to present the "shaven beard" appearance, and inflammation may involve much of the lining membrane of the bowel, especially the summits of the intestinal folds in the lower ileum and head of the colon about the region of the ileo-cecal valve. The mucous membrane is thickened and softened, and often small patches of ulceration are found, with enlarged intestinal glands and follicles, and even inflammatory enlargement of the mesenteric glands. Microscopically, the evidences of inflammatory action are found in cell-multiplication, vessel-dilatation, and tissue thickening in the acute cases as well as in those which run a more chronic course.

Hypostatic pulmonary congestion is also a nearly constant feature of this prostrating disease, and is due to circulatory failure. The interesting field of cerebral morbid anatomy belongs, more properly, to the field of another essayist.[1]

### TREATMENT OF SUMMER DIARRHEA OF CHILDREN.

BY DELANCEY ROCHESTER, M. D.

In speaking of that part of the subject under discussion this evening which has been allotted to me,—the Treatment of Summer Diarrhea of Children,—it is necessary, even at the risk of some repetition, to refer briefly to the causes which lead up to that dire condition of the bowels which carries off so many of our children under three years of age.

After a careful study of a limited number of cases in my own practice, and quite an extended reference to the literature on the subject,—and there is a great plenty of it,—I have come to the conclusion that the chief causal factors, aside from any hereditary constitutional taint, are: first, improper food; second, heat; third, impure air; fourth, bacterial infection.

Of course, the proper treatment of any disease, when its cause is known, is to remove that cause. Therefore, our first duty is to search for the cause in any given case. The causes enumerated above may act singly, or combined in groups of two, three, or four, as causal factors. Whatever the cause, it is, in my opinion, best to begin the

1. The MS. on this branch of the subject was not presented for publication.—Ed.

treatment of all these cases with a purge; and for that purpose there is nothing better than castor oil; the dose is comparatively small, it goes down easily, and is generally retained. A very palatable preparation of castor oil may be made in the following manner:

| | |
|---|---|
| ℞—Ol. Ricini | ℥ ii. |
| Ol. menth. pip. | m. v. |
| Sodii carb. exsiccat | gr. x. |
| Aquæ puræ | ℥ i. |

Dissolve the soda carbonate exsiccat in water, add the oils, and shake violently.

This makes a very thin emulsion, containing two-thirds castor oil, and is quite acceptable to most children. In those cases where castor oil is rejected, calomel is an excellent substitute. In children over three years old, it is often better than castor oil.

The rationale of the purge is that it cleans out the entire intestinal tract, removing all particles of undigested food, bacteria, or other irritating substances that may be present. After the action of the purge, if the first of the causal factors mentioned is the one at fault, the substitution of a proper food is often all that is necessary to restore the child to its place in the ranks of marching humanity.

But what is the proper food?

That is a question that requires careful consideration; and each case must be studied closely to learn what suits it best.

In the study of the patient, the pulse is of little value, because, generally, as soon as touched, the child jerks away its hand, or struggles so that its pulse beats very rapidly. The temperature is of more value, but it, too, is not nearly of as great significance as in the adult. Children will sometimes have a temperature that in the adult would necessarily be fatal, and still recover. The rectum is the most satisfactory place to take the temperature of the child.

But, in intestinal disturbances, there is always at least a congestion of the rectum, so that we often have a certain amount of local heat that must not mislead us. In these troubles, the thermometer in the rectum will often record 107° F., and not infrequently as high as 109° F., without fatal issue.

As the pulse and temperature, then, are not reliable guides, we must note the facial expression, the decubitus in sleep, whether restless or stupid, etc.; but, above all, the physician should personally examine the stools and urine of the child, and not rely upon the reports of the mother or nurse. In that way only can he tell whether the baby's food is properly digested, or is passed through the bowel in an undigested, or partially digested, condition, and whether there are any abnormal contents in the stool, such as blood, pus, worms, etc. If the child is vomiting, the vomited matter should also be carefully inspected.

Having determined that the food is not agreeing with the child, we return to our search for the best food. In the first year, the mother's milk is, of course, the best, and should be returned to if possible. After the first year, some other milk should form the chief, and for a time after the acute attack, the sole article of diet.

I shall not dilate upon the advantages of ass's, mare's, or goat's milk, as they are all more or less inaccessible. The question that confronts us, then, is how to give cow's milk to an infant under three years of age.

The first answer to this question is, Be sure of your cow, that she is not diseased, that she receives proper food, and is not kept constantly shut up in a stable. If all these conditions are complied with, then be sure that the milk is not carried too far, and that the vessels in which it is placed are kept scrupulously clean. Then, as soon as the milk arrives, dilute it with the proper amount of water, add a little cream and lime water, and sterilize it. The amount of dilution varies with the age of the child and its digestive power. That is the best food for infants in institutions, and in a very limited number of families in private practice. The reason that this number is so greatly limited is, in the first place, that, as a rule, we cannot be sure of our cows; and, in the next place, that we cannot rely upon the sterilization being performed in a thorough manner. Explain the reasons as carefully as we will, it is, nevertheless, a lamentable fact that people who think nothing of spending several hours over the preparation of their own foods, are often very loth to give even half an hour to the preparation of the child's food. Therefore, in the majority of cases, even where we are sure of the character of the milk, sterilized milk is out of the question. We must look for some other substitute. Provided that the milk supply is good, the food suggested by Dr. Arthur V. Meigs some years ago is a very excellent substitute. It consists of—

| | | |
|---|---|---|
| Cream (14–16 per cent. fat) . . . . . . . . . . . . . | 2 | volumes. |
| Cow's milk . . . . . . . . . . . . . . . . . . . . . . | 1 | " |
| Lime water . . . . . . . . . . . . . . . . . . . . . . | 2 | " |
| Sugar water . . . . . . . . . . . . . . . . . . . . . | 3 | " |

The sugar water consists of sugar of milk, ʒi. gr. viss. to water ℥i.

This combination is more suitable for children under one year of age, being made in imitation of human milk. As the child grows older, the proportionate amount of milk may be properly increased.

For a child over a year old, we may use cane sugar instead of sugar of milk. The food for a child should have cow's milk as its basis, diluted more or less, according to the age of the child. If diluted much, a certain amount of cream and sugar should be added to bring up the proportion of those two constituents, and the milk should be rendered alkaline by the use of lime water. In cases of weak digestive power, partially digested milk is useful. But, unfortunately, in the vast majority of cases in the city, good pure cow's milk cannot be obtained, and we must look for some substitute. In this search for a substitute, we must bear in mind that in childhood, as well as in adult life, the maxim holds good that, "What is one man's food is another man's poison." So we will not be able to find one food that will be applicable to all cases.

Condensed milk, diluted according to the age of the child, with lime water added, and, when procurable, good sweet cream, forms a very good food. For a child under one year of age, the following proportions are good, making a close approximation to human milk:

| | |
|---|---|
| Condensed milk (Eagle brand) | 1 volume. |
| Cream (14–16 per cent. fat) | 1 " |
| Lime water | 1 " |
| Boiled water | 4 " |

The objections to this combination are that most of its sugar is cane sugar, and the cream has to be obtained from dealers, and so may be adulterated or otherwise unwholesome.

There are two of the baby foods on the market which I think are useful and especially to be recommended during Summer, because they are both in the form of a dry powder, and need mixing with water only to prepare them for the child. These preparations are "Carnrick's Soluble Food," and "Malted Milk." With the former I have had quite a large experience, with almost uniform success, only very occasionally finding a child that would not take it. Over a year ago I introduced its use into the nursery at the Erie County Almshouse, and last Sunday, July 13th, I had occasion to go out there, and found them still using it, almost to the exclusion of every other form of food. With malted milk I have had a much more limited experience, though the results have been equally good. However, I prefer to recommend the former, because its manufacturers publish its formula, while the Malted Milk Co. keeps its formula secret.

Other foods for infants, which are sometimes useful, are scraped raw beef, fresh beef juice, and Liebig's extract of beef.

The farinaceous foods had better be avoided, except in the case of children of three or more years of age, when they may be useful adjuvants to the meat, or, in some cases, be used to the exclusion of all else. For example, rice, or rice flour, is a very useful article of diet in the diarrhea of children over three years of age. The following mixture is sometimes of use in cases which tend to become chronic:

| | |
|---|---|
| Grated chocolate . . . . . . . . . . . . . . . . . . . . . . | 1 lb. |
| Arrow root . . . . . . . . . . . . . . . . . . . . . . . . . | ½ lb. |
| Sifted sugar . . . . . . . . . . . . . . . . . . . . . . . . | ¾ lb. |
| Rice flour . . . . . . . . . . . . . . . . . . . . . . . . . | 2 oz. |

Mix all well together. Blend a tablespoonful of this mixture with a tablespoonful of water and then add one-half pint of boiling milk, stirring during the addition.

I have found this a very useful food, both for children and for adults who have diarrheal tendencies. The mixture is called "Racahout." What the name means, and where it originated, I have not the slightest idea.

Above all things, in the feeding of children, the *absolute cleanliness* of the vessels used must be insisted upon; and, to quote from a paper that I read before the "Physicians' Club," in December, 1887, "whatever the food we employ, let the amount given be proportionate to the capacity of the child's stomach. It cannot be too strongly impressed upon the mind of the mother that she must not give her child too much food. There is always greater danger from overfeeding than from underfeeding." The child's stomach, at birth, will hold from one ounce to one ounce and a half, according to the size of the child, and its capacity increases at the rate of about one ounce a month for the first year. After that time, reliable statistics are wanting. Moreover, the child should not be fed too often; never oftener than once in two hours, and only so often when one or two ounces are given at a time. But improper food is not the only causal factor in the production of Summer diarrhea of children. Excessive heat, impure air, and bacterial infection each play a contributing part.

Without entering into any discussion of the mode of action of any of these factors—a task which belongs to the preceding papers—I will merely outline the mode of treatment which has been most successful in my own hands.

In the first place, if the heat of the child is excessive, sponging with tepid or rather cool water, ice-water applications to the head, and occasionally to the spine, have been followed by amelioration of the distressing restlessness and delirium that frighten the attendants and weaken the child.

With the so-called antiseptic treatment of children's diarrhea, namely, the use of bichloride of mercury, sodium salicylate, naphthalin, etc., after the purge, I have had no success.

There are two prescriptions that I took from an article by J. Lewis Smith, of New York, which appeared three or four years ago in one of the journals, that have stood me in good stead in many a case. They are useful only in the first part of an attack, and are to be used in cases of vomiting and diarrhea due to indigestion, accompanied by acidity. For the first twenty-four hours, a powder consisting of—

| | |
|---|---|
| Pulv. ipecac | gr. $\frac{1}{24}$ |
| Pulv. rhei | gr. $\frac{1}{6}$ |
| Sodii bicarb. | gr. 1 |

is to be given once in four hours.

This alone often sets things all right. If, however, it does not, there is no use pushing it any further. The other prescription then comes into play:

| | |
|---|---|
| ℞—Tinct. opii deodorat. | gtt. xvi. |
| Bismuthi subnitratis | ʒ ii. |
| Syr. simplicis | ℥ ss. |
| Mist. Cretæ | ℥ iss. |

M. et Sig.—One teaspoonful every three or four hours.

In the more serious cases, with watery discharges, minute doses of calomel will often change the character of the movements and lessen their frequency. When there is much pain and straining, a little opium combined with the calomel has a very happy effect.

It is well not to check these disturbances too suddenly; but, on the other hand, we must not let them run away with us, and astringents have a proper place in their treatment. Of the vegetable astringents, logwood, kino, catechu, and tannic acid are highly recommended, but, in my hands, the mineral astringents have proved more beneficial, and of them the dried sulphate of iron the most useful.

In cases of hemorrhage, alum is probably the best drug at our command. In one very severe case, with bloody mucous discharges, I had a very beneficial result from injecting high up into the bowel a solution of silver nitrate gr. ii. to ℥ iv. of water, and, after this had been retained for half an hour, washing out with a larger injection of borax water. Under the use of this alone three or four times a day, in two days the tenesmus ceased, the mucus disappeared from the stools, and the stools themselves became much reduced in frequency.

Stimulants are seldom needed, but, when needed, they should be given in the form of champagne or brandy, in small quantities frequently repeated. A pint of iced champagne given in the course of twenty-four, or even twelve hours, to a child two years of age, will sometimes save its life.

In closing my remarks on treatment, I wish to reiterate what I said at first, that I believe that all cases of diarrhea in children should be treated in the beginning with a purge of castor oil or calomel, except cases in which violent hemorrhage from the bowels is taking place.

These are best treated with alum and stimulants, but these remedies should be followed by castor oil as soon as their object is accomplished. Then the diet should be carefully regulated, and such other steps taken in the way of medication as are indicated by the case in hand.

